# Parameter estimation and mathematical modeling for the quantitative description of therapy failure due to drug resistance in gastrointestinal stromal tumor metastasis to the liver

**DOI:** 10.1371/journal.pone.0217332

**Published:** 2019-05-30

**Authors:** Patricio Cumsille, Matías Godoy, Ziomara P. Gerdtzen, Carlos Conca

**Affiliations:** 1 Group of Investigation in Tumor Angiogenesis (GIANT), Group of Research and Innovation in Vascular Health (GRIVAS Health), Basic Sciences Department, Universidad del Bío-Bío, Chillán, Chile; 2 Centre for Biotechnology and Bioengineering (CeBiB), Santiago, Chile; 3 Department of Chemical Engineering, Biotechnology and Materials, University of Chile, Santiago, Chile; 4 Department of Mathematical Engineering (DIM) and Center for Mathematical Modeling (CMM), University of Chile, (UMI CNRS 2807), Santiago, Chile; UCLA, UNITED STATES

## Abstract

In this work we develop a general mathematical model and devise a practical identifiability approach for gastrointestinal stromal tumor (GIST) metastasis to the liver, with the aim of quantitatively describing therapy failure due to drug resistance. To this end, we have modeled metastatic growth and therapy failure produced by resistance to two standard treatments based on tyrosine kinase inhibitors (Imatinib and Sunitinib) that have been observed clinically in patients with GIST metastasis to the liver. The parameter identification problem is difficult to solve, since there are no general results on this issue for models based on ordinary differential equations (ODE) like the ones studied here. We propose a general modeling framework based on ODE for GIST metastatic growth and therapy failure due to drug resistance and analyzed five different model variants, using medical image observations (CT scans) from patients that exhibit drug resistance. The associated parameter estimation problem was solved using the Nelder-Mead simplex algorithm, by adding a regularization term to the objective function to address model instability, and assessing the agreement of either an absolute or proportional error in the objective function. We compared the goodness of fit to data for the proposed model variants, as well as evaluated both error forms in order to improve parameter estimation results. From the model variants analyzed, we identified the one that provides the best fit to all the available patient data sets, as well as the best assumption in computing the objective function (absolute or proportional error). This is the first work that reports mathematical models capable of capturing and quantitatively describing therapy failure due to drug resistance based on clinical images in a patient-specific manner.

## Introduction

Gastrointestinal stromal tumors (GISTs) are the most common mesenchymal tumors of the gastrointestinal tract, with an incidence of 11-15 cases per million people per year. It is estimated that 40-50% of GISTs are biologically malignant, and have spread to the liver or peritoneum at the time of diagnosis or primary surgery [1]. One of the molecular characteristics of these neoplasms is a gain of function mutation in the receptor tyrosine- kinase protein (KIT) (75-80% of cases) or the homologous receptor tyrosine kinase, platelet-derived growth factor receptor alpha (PDGFRA), accounting for 85-90% of gastrointestinal stromal tumors [2]. In addition to the primary mutation, secondary mutations have also been identified in patients with advanced GIST pretreated with tyrosine kinase inhibitor. To date, ten different molecular subsets of GIST with different molecular alterations have been reported [1]. For most cases of resectable/non-metastatic GISTs cases treatment involves surgical resection, and tyrosine kinase inhibitor (TKI) therapy may be utilized to reduce tumor size before resection.

For metastatic or non-resectable GISTs the treatment of choice is TKI therapy [2]. Imatinib is utilized as the first-line drug as it acts best on the most frequent KIT mutations. In 85% of the cases Imatinib can control the metastatic disease during a 20-24 months period. After resection, adjuvant Imatinib therapy has also been found to improve recurrence-free and overall survival. However, as reported by Blay [3], Imatinib resistance is frequently observed. This resistance is associated to the specific exon where the mutation occurs.

Sunitinib or Sorafenib is a tyrosine kinase inhibitor molecule that targets KIT and has antiangiogenic effects, which is utilized for the treatment of advanced gastrointestinal stromal tumors in patients who fail Imatinib therapy. The treatment and prognosis of patients with gastrointestinal stromal tumors depends on the oncogenic kinase mutations that caused it, and the utilization of specific molecular therapies that inhibit this molecular defect. However, GISTs include several different molecular subtypes that vary in their response to kinase inhibitors. Therefore, it is crucial to correctly identify the tumor’s response to treatment in order to assess a suitable treatment timely. For clinicians, one critical challenge is to optimize cancer treatments, and to determine the more adequate time to switch from the first-line to the second-line treatment for increased overall survival. To do this, relapse time estimation is a critical issue [4]. Given that prognosis and sensitivity to treatment are patient-dependent, we aim at developing patient-dependent mathematical models based on medical images of liver metastasis. We focus on locally advanced GISTs to quantitatively describe for each patient the time of emergence of mutations in cancer cells, as well as the relapse time after the first-line and the second-line treatments.

Mathematical modeling has been extensively utilized in recent years to shed light on cancer progression, emphasizing the issue of rendering patient specific models (see [4–6]). However, due to the complexity of the processes involved in all the stages of neoplastic growth, mathematical models must be limited to a few phenomena, and are therefore a simplification of what occurs in the biological system. The key task is then to develop mathematical models that are able to capture most of the relevant features of cancer progression. In this type of models parameter estimation becomes an important problem that requires a rational experimental design and clinical data collection. Even though mathematical models of GIST metastasis to the liver, growth and therapy failure associated to drug resistance are available, the latter has not been described quantitatively using mathematical models and considering clinical images in a patient-specific manner. Typically, mathematical models utilized for clinical applications in this field do not consider the spatial aspect of tumor growth. They are often parametrized using statistical methods and may provide a prognosis of tumor volume, among other important aspects [5]. Considering this, we aim at developing patient-specific models that can capture the evolution of the metastatic tumor, as well as quantitatively describe and explain therapy failure due to drug resistance. Clinical follow-up to monitor the disease evolution is mainly performed by CT scans. Using observations from these medical images we applied a hybrid approach [5] to develop patient-specific mathematical models with the purpose of quantitatively describing therapy failure due to drug resistance in the case of GIST metastases to the liver. We built a general modeling framework consisting of a nonlinear system of ordinary differential equations (ODE) that take into consideration the volume of the sensitive and resistant tumor cells to conventional treatments based on tyrosine kinase inhibitors in a different manner, as it has been addressed in [4]. From a mathematical modeling point of view, one of the strengths of the work presented here is the response to treatment modeling. We describe GIST metastases to the liver, growth and therapy failure due to drug resistance, following a modeling strategy that considers three different cell populations, and the model was developed from mass balances for these cell populations, describing tumor growth, death and angiogenesis. Two possible treatments and outcomes were considered: the first treatment outcome is a cytotoxic effect, as associated to the treatment with Imatinib, and the second treatment has both cytotoxic and anti-angiogenic effects as observed with Sunitinib or Sorafenib. Three different homogeneously distributed proliferative cell populations are utilized to describe treatment resistance: one population that is sensitive to both treatments, one that is only sensitive to the second treatment and a third cell population that is resistant to both treatments. A simple representation of angiogenesis, which is crucial to explain metastatic growth, has also been considered. Model parameters represent biologically meaningful quantities including the growth and death rate of each cell population considered, nutrient availability, among others. The general model is highly complex as it contains nonlinear terms that allow the representation of different biological responses. Five different model variants were analyzed, with parameters that represent specific cell population distributions and sensitivity to treatment scenarios.

Parameter estimation involves solving the inverse problem: given a model and measurements of some state or output variables, the parameters that characterize the system, *i.e*. those producing a good fit of the model with the data, need to be identified [7]. This problem is difficult to solve since no unique analytical or numerical solution is available [6]. Even if a unique solution was available, good initial values would be required to compute a good parameter estimate, since a minimization to match the experimental data with the model solution should be solved, and optimization solvers require initial parameter values close to the actual ones to get accurate numerical solutions. For the type of biological system studied in this work, obtaining good initial parameter values can be difficult given that the *a priori* knowledge on the system is limited. Therefore, finding the set of parameters that can be reliably estimated for a given model and a set of empirical observations will require a parameter identification process [6, 7]. We implemented a parameter identification method using the proposed nonlinear ODE models described above that can represent tumor growth and therapy failure due to drug resistance, and therefore an accurate fit for patient data with quantitative descriptive capabilities can be expected. Given the complexity of these models, theoretical as well as numerical resolution of the parameter estimation problem is difficult since data are sparse and only one partial combination of model variables is measured experimentally. A practical identifiability approach was conducted on each of the five model variants proposed and they were compared from a goodness of fit point of view.

The work presented here is the first to quantitatively describe GIST therapy failure due to drug resistance based on clinical images, by using mathematical models. This is highly relevant given the limitations on data availability, and the observation of only a partial combination of the proposed models variables. We expect our work to provide an insight on tumor response to treatment that may contribute to the design of new therapeutic strategies for minimizing drug resistance.

This article is organized as follows. In the General modeling framework section we introduce mathematical modeling of metastatic growth and drug resistance in the case of GIST metastasis to the liver. In the Practical identification approach section we develop a practical identifiability approach where we describe the techniques utilized to solve the parameter estimation problem of our proposed models, the goodness of fit criteria required to evaluate and compare them, and provide a manner to assess the statistical assumptions in order to achieve the best parameter estimation results. The Results and discussion section is devoted to showing and discussing in detail the results obtained, and we present our conclusions in the Summary and conclusions section.

## Materials and methods

### General modeling framework

In a previous work, a model consisting of a system of nonlinear partial differential equations that simulates tumor drug resistance as well as spatial heterogeneity of tumor growth, was developed [4]. However, due to its complexity, it only reproduced the behavior observed in GIST metastasis patients from a qualitative point of view. Other models for tumor growth reviewed in Cumsille *et al*. [5] have similar limitations. In a first attempt to address the issue of describing such features in a quantitative manner, we developed a general mathematical model based on mass balances for tumor cells, studied five model variants given by specific parameters associated to cell populations and response to treatments, and applied a practical identifiability approach to these models using empirical data for two patients. Both theoretical as well as numerical resolution of the associated parameter estimation problem are difficult due to the fact that empirical data are sparse, and only one partial combination of models variables, tumor area, is observed. In addition, it is not possible to obtain a good initialization of models parameters to solve the least squares problem associated to the inverse problem, since we do not have enough *a priori* information on the system. In particular, one cannot know neither the actual proportion of sensitive and resistant to treatments cells populations (at any time) nor their time of emergence.

The general model accounts for three different proliferative tumor cell populations, which are utilized to describe the resistance to treatments and they consider tumor growth in different manners. The tumor is described by means of three different proliferative tumor cell populations. One proliferative tumor cell population is sensitive to both treatments, another one is only sensitive to the second-line treatment, while the third one is resistant to both treatments. The general model also considers cell death and angiogenesis, which is a key factor in metastatic growth. Spatial aspects of tumor growth as well as a distinction between healthy and necrotic cell populations are not considered, allowing us to reduce the complexity and number of parameters of the model. This allows for a more manageable model from a parameter estimation point of view, while maintaining a sufficiently complex general model structure that can capture different possible tumor progression scenarios for the cancer studied.

#### Treatment description

As discussed in the Introduction, two treatments are considered: the first-line treatment, which will be denoted by *τ*_1_, is a specific tyrosine kinase inhibitor such as Imatinib, which has a cytotoxic effect on proliferative tumor cells; the second-line one, denoted by *τ*_2_, is a multi-targeted kinase inhibitor such as Sunitinib or Sorafenib, which has both cytotoxic and anti-angiogenic effects. In addition to the cytotoxic effect, it blocks the production of growth factors such as vascular endothelial growth factors and thus decreases the nutrient supply brought to the tumor. It is well known that cytotoxic drugs do not impact similarly all the metastatic cancer cells since resistant phenotype can appear in the proliferative cell population. Moreover, cancer cells can respond differently to hypoxia. To account for this, as in [4], we split the proliferative tumor cells, which volume is denoted by *P*, into three subpopulations where

- *P*_1_ denotes the volume of the proliferative tumor cells that are sensitive to *τ*_1_ and also to *τ*_2_;
- *P*_2_ describes the volume of the proliferative tumor cells that are resistant to *τ*_1_ and sensitive to *τ*_2_; and
- *P*_3_ stands for the volume of the proliferative tumor cells that are resistant to both treatments *τ*_1_ and *τ*_2_.

It is worth noting that we do not aim at describing the evolution of the tumor from an early stage of the GIST, but we only focus on the evolution of metastasis located in the liver. Therefore, based on clinical observations, it is relevant to consider that all three cell subpopulations are present when the GIST metastasis is detected.

#### General model and its variants

The general mathematical model proposed is written as a nonlinear ODE system, based on mass balance principle accounting for the volumes of the different proliferative tumor cells, as well as vascularization and nutrient supply through angiogenesis.

#### Volumes of the proliferative tumor cells

The volumes of the proliferative tumor cells obey a mass balance principle according to the following general equation:

$$\begin{array}{c} P_{i}^{\prime }=[\mu \left(M\right)-\delta \left(M\right)-\delta_{i}^{treat}\left(M\right)]P_{i},\;\text{for}\;i=1,2,3. \end{array}$$ (1)

In above equation, *μ*(*M*) and *δ*(*M*) denote cellular growth and death rates respectively. Both variables depend on *M*, which represents vascularization and nutrient supply, according to

$$\begin{array}{ccc} \mu (M) & = & \mu_{MAX}\frac{1+\tanh(R\left(M-M_{hyp}\right))}{2}\;, \end{array}$$ (2)

$$\begin{array}{cc} \delta (M)= & \delta_{MAX}\frac{1-\tanh(R\left(M-M_{hyp}\right))}{2} \end{array}$$ (3)

where *μ*_*MAX*_ and *δ*_*MAX*_ are the maximum growth and death rates of the tumor cells respectively, and *M*_*hyp*_ is the hypoxia threshold, below which nutrients can be considered limiting for cell growth. These expressions account for the fact that if nutrients are above the limiting threshold, *i.e*. *M* > *M*_*hyp*_, then *μ*(*M*) ≈ *μ*_*MAX*_ and *δ*(*M*) ≈ 0 and consequently tumor cells undergo proliferation. The functions *μ*(*M*) and *δ*(*M*) are regularized versions of the sigmoid Heaviside function. In this function, *R* is a numerical parameter that controls the function’s slope and was set to 5 in order to provide a smooth transition between the non-growth/maximum death rates and the maximum growth/non-death rates. An additional death term was added in Eq (1) to account for the effect of the first and second-line treatments *τ*_1_ and *τ*_2_. We have denoted by $\delta_{i}^{treat}\left(M\right)$ the death rate due to the treatments, which is related to the dose of drug delivered to the patient, among others factors. Note that the subscript *i* is to account for the fact that treatments may have a different effect in the different proliferative tumor cell subpopulations *i* = 1, 2, 3. The functions $\delta_{i}^{treat}\left(M\right)$ are defined by:

$$\begin{array}{cc} \delta_{1}^{treat}\left(M\right)= & [\delta_{1}\chi_{1}\left(t\right)+\delta_{2}\chi_{2}\left(t\right)]\;\left(\alpha +M\right), \end{array}$$ (4)

$$\begin{array}{ccc} \delta_{2}^{treat}\left(M\right) & = & \delta_{2}\chi_{2}\left(t\right)\left(\alpha +M\right), \end{array}$$ (5)

$$\begin{array}{ccc} \delta_{3}^{treat}\left(M\right) & = & 0. \end{array}$$ (6)

In above equations, we have denoted by

$$\begin{array}{c} \chi_{1}\left(t\right)=1_{\left\{t<T_{j}\right\}}\left(t\right)\;\left(\text{resp.}\;\chi_{2}\left(t\right)=1_{\left\{t\geq T_{j}\right\}}\left(t\right)\right) \end{array}$$ (7)

the characteristic function of treatment *τ*_1_ (resp. *τ*_2_), where *T*_*j*_ is the time at which physicians switch from *τ*_1_ to *τ*_2_ treatment for each patient *j* = 1, 2 considered in this work. Moreover, *δ*_*k*_ is the maximum death rate due to treatment *τ*_*k*_ for *k* = 1, 2.

Finally, the parameter *α* in Eqs (4) and (5) stands for a quantification of a basal vasculature, which is set to be 0 or 1, depending on whether the model variant considers this basal level or not.

#### Vascularization, nutrient supply and angiogenesis

In general Eq (1), the variable *M* describes two fundamental issues, vascularization and nutrient supply driven by tumor angiogenesis; see [5] for a detailed overview on tumor growth. It is worth noting that the second-line treatment effect has to be taken into account in these two related aspects.

Since the nutrients are supplied to the tumor by the vascularization, as a simple way to represent both aspects, only one variable is utilized to describe the nutrient concentration and the vascularization; see [4]. Let us denote by *M* this variable, which is governed by a mass balance principle:

$$\begin{array}{c} M^{\prime }=\gamma \frac{\delta (M)}{\delta_{MAX}}{\left\{\left(1-\nu \chi_{2}\left(t\right)\right)\left(P_{1}+P_{2}\right)+\zeta P_{3}\right\}}^{2/3}-\beta \left(M\right)MP. \end{array}$$ (8)

Since all three proliferative tumor cell subpopulations 1, 2 and 3 produce angiogenic factors, nutrient availability increases. This is represented in Eq (8) by means of an increase of *M* when the system is below the hypoxia threshold *M*_*hyp*_ as given by the term *γ* ⋅ *δ*(*M*)/*δ*_*MAX*_, where *γ* represents the angiogenic capacity of the proliferative tumor cell population that leads to an increase in nutrients associated to the additional vasculature induced by the tumor. The effect of the angiogenesis is reduced by *τ*_2_, which has both cytotoxic and anti-angiogenic effects, therefore it acts on population volume *P*_1_ + *P*_2_ that are sensitive to this treatment. The effect of *τ*_2_ in Eq (8) is represented directly by means of the term *νχ*_2_(*t*) that affects *P*_1_ + *P*_2_ causing *M* to decrease, or indirectly by a relative increase of the resistant population volume *P*_3_ with respect to volume *P*_1_ + *P*_2_, where the dimensionless parameter *ν* corresponds to the anti-angiogenic effect of *τ*_2_, and the dimensionless parameter *ζ* represents the relative increase of *P*_3_. It is worth noting that the exponent 2/3 in the first term at the right-hand side of Eq (8) accounts for the fact that nutrient availability must be proportional to the tumor cells’ surface. In the right-hand side term, *β*(*M*) denotes the rate of nutrients consumption, which can be considered as constant, *i.e*. *β*(*M*) = *β*, where *β* stands for a constant parameter, or can be considered as dependent on the normalized growth rate *μ*(*M*)/*μ*_*MAX*_ as *β*(*M*) = *β* ⋅ *μ*(*M*)/*μ*_*MAX*_. In the latter case, the role of the term *μ*(*M*)/*μ*_*MAX*_ allows to prevent nutrient consumption by the tumor cells, *β* ⋅ *μ*(*M*)/*μ*_*MAX*_ ⋅ *MP*, from becoming too large. The normalized growth rate *μ*(*M*)/*μ*_*MAX*_ in the term *β*(*M*) is highly meaningful, since when the proliferative tumor cell population increases at high rates, *i.e*. when *μ*(*M*) ≈ *μ*_*MAX*_, then the nutrient consumption by the tumor cells *β* ⋅ *μ*(*M*)/*μ*_*MAX*_ ⋅ *MP* ≈ *βMP* leading to a high nutrient consumption; while when the proliferative tumor cell population stalls, *i.e*. when *μ*(*M*) ≈ 0, then nutrient consumption by the tumor cells is also reduced, *β* ⋅ *μ*(*M*)/*μ*_*MAX*_ ⋅ *MP* ≈ 0.

Table 1 provides a detailed description of the general model’s variables and parameters, as well as the notation utilized.

**Table 1:** Variables, functions and parameters for the general model.

Name	Description	Unit
*τ*_1_	First-line treatment, Imatinib	–
*τ*_2_	Second-line treatment, Sunitinib or Sorafenib	–
*P*_1_	Volume of the proliferative cells sensitive to *τ*_1_ and *τ*_2_	mm^3^
*P*_2_	Volume of the proliferative cells resistant to *τ*_1_ and sensitive to *τ*_2_	mm^3^
*P*_3_	Volume of the proliferative cells resistant to *τ*_1_ and *τ*_2_	mm^3^
*M*	Normalized nutrient concentration	–
*μ*(*M*)	Growth rate	d^−1^
*δ*(*M*)	Death rate	d^−1^
*μ*_*MAX*_	Maximum growth rate	d^−1^
*δ*_*MAX*_	Maximum death rate	d^−1^
*χ*_1_(*t*)	Characteristic function for *τ*_1_	–
*χ*_2_(*t*)	Characteristic function for *τ*_2_	–
$\delta_{i}^{treat}\left(M\right)$	Death rate due to the treatments	d^−1^
*δ*_1_	Maximum death rate due to *τ*_1_	d^−1^
*δ*_2_	Maximum death rate due to *τ*_2_	d^−1^
*α*	Basal vasculature index	–
*M*_*hyp*_	Hypoxia threshold	–
*γ*	Tumor angiogenic capacity	mm^−2^⋅d^−1^
*β*(*M*)	Nutrient consumption rate	mm^−3^⋅d^−1^
*ν*	Decrease of *P*_1_ + *P*_2_ due to antiangiogenic effect of *τ*_2_	–
*R*	Regularizing parameter for the approximate Heaviside function	–
*ζ*	Relative increase of *P*_3_ due to the antiangiogenic effect of *τ*_2_	–

#### Summary of the general model, their vector formulation and its variants

Gathering the general Eq (1) for *P*_*i*_ and general Eq (8) for *M* we obtain the following ODE system:

$$\begin{array}{ccc} P_{i}^{\prime } & = & [\mu \left(M\right)-\delta \left(M\right)-\delta_{i}^{treat}\left(M\right)]P_{i},\;\text{for}\;i=1,2,3. \end{array}$$ (9)

$$\begin{array}{ccc} M^{\prime } & = & \gamma \frac{\delta (M)}{\delta_{MAX}}{\left\{(1-\nu \chi_{2}\left(t\right))\left(P_{1}+P_{2}\right)+\zeta P_{3}\right\}}^{2/3}-\beta \left(M\right)MP, \end{array}$$ (10)

The general model proposed in this work is written under the form given by general Eqs (9) and (10). We analyzed five different variants resulting from letting vary or fixing specific parameters as indicated in Table 2.

**Table 2:** Model variants proposed.

Model Variant №	*α*	*ν*	*ζ*	*β*(M)
1	0	Variable	Fixed to 1	*β*
2	0	Fixed to 1	Variable	*β*
3	1	Variable	Fixed to 1	*β*
4	1	Variable	Fixed to 1	*β* ⋅ *μ*(*M*)/*μ*_*MAX*_
5	0	Variable	Fixed to 1	*β* ⋅ *μ*(*M*)/*μ*_*MAX*_

The model variants proposed in Table 2 account for different physiological scenarios that may occur for a GIST. Variants 1 and 2 do not consider a basal vasculature *α* associated to the tumor, and a constant nutrient consumption rate *β* is assumed. The difference between both cases is the representation of the effect of the second-line treatment *τ*_2_; for variant 1 it is represented as a acting directly on the sensitive tumor cells (variable *ν*), whereas for variant 2 its effect is considered to be indirect by increasing the resistant tumor cell population relative to the sensitive ones (variable *ζ*). Variants 3 and 4 consider a basal vasculature (*α* = 1) and the second-line treatment is represented as a direct effect on the sensitive tumor cells (variable *ν*). The difference between these two variants is the nutrient consumption rate description, which is assumed to be constant for variant 3 (*β*) while for variant 4 it is considered to be proportional to the normalized growth rate (*β*(*μ*)). Variant 5 is similar to variant 4, but with a basal vasculature (*α* = 1). These five model variants were selected in order to test for the possible physiological mechanisms involved in therapy failure observed in patients with drug resistance. For simplicity, model variants 1 to 5 in Table 2 will be referred to hereafter as Models 1 to 5.

The same initial conditions were considered for all the models analyzed:

$$\begin{array}{c} M\left(0\right)=M^{0j},\;P_{i}\left(0\right)=P_{i}^{0j}\;\text{for}\;\text{each}\;i=1,2,3\;\;\text{and}\;\text{for}\;\text{each}\;\text{patient}\;j=1,2. \end{array}$$

Finally, all the models previously described can be written under the vector form:

$$\begin{array}{ccc} U^{\prime } & = & F(t,U,\theta ), \end{array}$$ (11)

$$\begin{array}{ccc} U(t_{0}) & = & U_{0}, \end{array}$$ (12)

where *U*(*t*) corresponds to the vector of state variables of the system, given by *U*(*t*) = [*P*_1_(*t*), *P*_2_(*t*), *P*_3_(*t*), *M*(*t*)] for all *t* ∈ [*t*_0_, *t*_*f*_], where *t*_*f*_ > *t*_0_ is a sufficiently large time as to let the system evolve, $U^{0}=[P_{1}^{0},P_{2}^{0},P_{3}^{0},M^{0}]$ is the initial state of the system, and the function *F* represents the right-hand side of the corresponding model variant. For instance, for Model 1 one has:

$$\begin{array}{c} F\left(t,U,\theta \right)=[\begin{array}{c} {}[\mu (M)-\delta (M)]P_{1}-[\delta_{1}\chi_{1}\left(t\right)+\delta_{2}\chi_{2}\left(t\right)]MP_{1}\\ {}[\mu (M)-\delta (M)]P_{2}-\delta_{2}\chi_{2}\left(t\right)MP_{2}\\ {}[\mu (M)-\delta (M)]P_{3}\\ \gamma \frac{\delta (M)}{\delta_{MAX}}{\left\{\left(1-\nu \chi_{2}\left(t\right)\right)\left(P_{1}+P_{2}\right)+P_{3}\right\}}^{2/3}-\beta MP \end{array}]\;, \end{array}$$ (13)

where $\theta ={\left(\mu_{MAX},\delta_{MAX},\delta_{1},\delta_{2},M_{hyp},\gamma ,\beta ,\nu \right)}^{t}\in R^{8}$ are the parameters of Model 1.

#### Modeling capabilities

**Fig 1:**
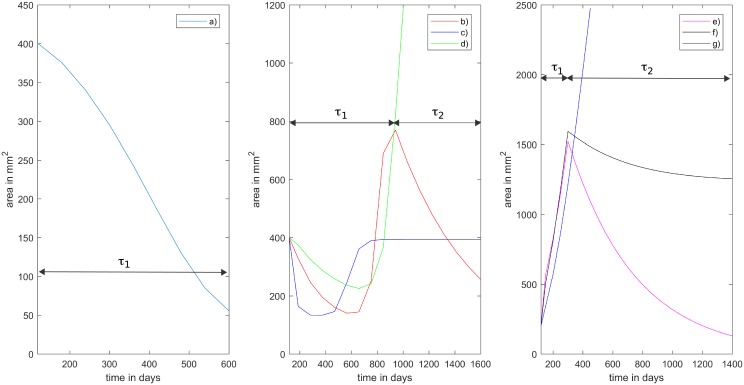
Modeling capabilities. Our general model is able to reproduce the different scenarios reported by physicians. Left: a) *τ*_1_ effective, no *τ*_2_ applied. Center: *τ*_1_ is applied then switched to *τ*_2_, b) *τ*_1_ partially effective, tumor regrows and *τ*_2_ reduces tumor size; c) *τ*_1_ is effective, *τ*_2_ is effective in maintaining tumor size; d) *τ*_1_ is effective, *τ*_2_ is ineffective. Right: *τ*_1_ is ineffective in reducing or maintaining tumor size and it is switched to *τ*_2_, e) *τ*_2_ is effective in reducing tumor size; f) *τ*_2_ only stabilizes the tumor size; g) *τ*_2_ is completely ineffective.

**Fig 2:**
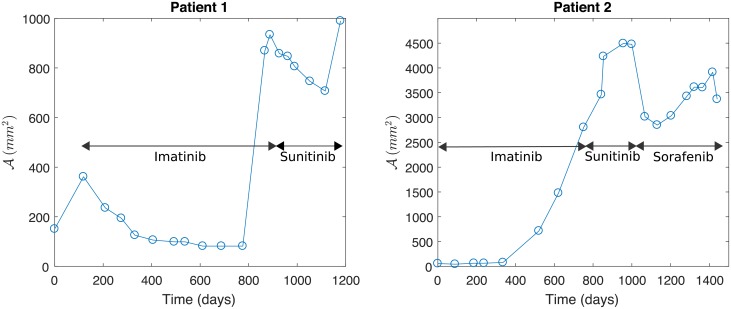
Data sets from two patients representing the two typical metastasis evolution patterns under a drug resistance scenario. Profiles depict tumor area A (in mm^2^) vs. time *t* (in days). Left: Metastasis is controlled by Imatinib, which is delivered from day *t* = 119, before a first relapse. Then, treatment with Sunitinib is efficient before a second relapse. Right: Treatment with Imatinib, delivered from day *t* = 0 to *t* = 845, is ineffective. Then, the treatment with Sunitinib, delivered from day *t* = 845 to *t* = 1049, and Sorafenib, delivered from day *t* = 1049 to *t* = 1600, is relatively effective until a relapse is observed.

By varying the parameter vector $\theta ={\left(\mu_{MAX},\delta_{MAX},\delta_{1},\delta_{2},M_{hyp},\gamma ,\beta ,\nu \;\text{or}\;\zeta \right)}^{t}\in R^{8}$, the proposed models can account for several possible tumor progression scenarios that have been reported by physicians [4]. Fig 1 curve a) depicts the case where the first-line treatment *τ*_1_ is applied starting on day 119 and is completely effective, so there is no need to consider the second-line treatment *τ*_2_. Patient 1 received no treatment from day 0 to day 119. This is accounted for in the simulation (see Fig 2, left) where tumor growth is observed initially and day 119 is the initial day of treatment for patient 1. Fig 1 (center) depicts the case where *τ*_1_ is applied from day 119 until day 867, and it is then switched to *τ*_2_. Several possible responses may be observed in this context: in curve b) *τ*_1_ initially works but the tumor regrows and the subsequent application of *τ*_2_ successfully reduces the tumor size; in curve c) *τ*_1_ works as before, whereas *τ*_2_ only keeps the tumor size fixed without reducing it; finally, in curve d) *τ*_1_ behaves as before whereas *τ*_2_ is ineffective since the tumor size explodes. Fig 1 (right) depicts the scenario in which *τ*_1_ is applied from day 119 until day 300, then it is switched to *τ*_2_ when *τ*_1_ becomes ineffective (tumor size increases under its action); we can observe that only in curve e) *τ*_2_ is effective as it reduces the tumor size; in curve f) it only stabilizes the tumor size, and in curve g) it is completely ineffective.

Similar capabilities have been described for the partial differential equation (PDE) model reported in [4].

### Practical identification approach

In this section we describe the parameter identification problem, as well as the methods required to solve it. In addition we describe goodness of fit criteria to compare all the proposed model variants, summarized in Table 2. These criteria allow us to assess the fit performance of the different models.

It is worth noting that we do not aim to prove results on structural identifiability, since in general parameter identification problems are ill-posed; in particular they are unstable with respect to data noise. Structural identifiability provides *a priori* information about model parameters, yielding a necessary but not sufficient condition for successful parameter estimation from real data, which are typically incomplete and noisy [6]. In our case, structural identifiability is not trivial to prove since our proposed models, summarized in Table 2, contain non-rational functions that are difficult to handle in such analysis (see functions in Eqs (2) and (3)). In the works by Miao and Wu [7, 8] the proposed methods rely strongly on the linearity (or, at most rational expressions) in order to provide identifiability assertions. In addition, in our system only one partial linear combination of the model variables is observed, since the actual proportion of the sensitive/resistant to treatments tumor cells populations is not known. On the other hand, even for simpler models in the context of tumor growth, results on non-uniqueness for the parameter identification problem have been proven; see [9]. We have observed numerically that our model is not stable with respect to the parameters, since there are two different set of parameters that yield very close model responses in a time interval, but which represent two different long-term behaviors. Fig 1 depicts several tumor responses to therapies that have been reported by physicians [4]. In particular, in Fig 1 (right) three different scenarios show almost the same behavior during the first 400 days, however, their long-term behaviors are significantly different. When comparing the three curves for the first 400 days only, we could infer that the corresponding sets of parameters should be almost the same therefore implying model stability, which is not the case. This shows that it is not possible to predict the complete evolution of a solution by knowing only its early behavior. Discussion on this feature for a PDE model can be found also in [4].

We devised a practical identifiability approach to be applied to this system by addressing two specific tasks. First, in order to solve the parameter estimation problem, *i.e*. to find the parameters (summarized in Table 2) that fit our proposed models to the data; and then to compare the goodness of fit to data of our proposed models. In what follows we explain in detail each of these tasks.

#### Parameter estimation problem

To perform parameter estimation, empirical data obtained from the CT scans of two different patients, which are representative of the possible scenarios where drug resistance is observed for the disease, are utilized. The first scenario accounts for 85% of cases, where *τ*_1_ with Imatinib controls the metastatic tumor during a more or less long period of around 20-24 months. After this, physicians change to the second-line treatment *τ*_2_ with Sunitinib or Sorafenib. In the second scenario, representing the remaining 15% of cases, an Imatinib resistance due to a secondary mutation in the receptor tyrosine- kinase protein gene is observed early on after treatment is started. Representative data from patients for these two possible scenarios are shown in Fig 2. The source data for constructing Fig 2 are provided in S1 and S2 Tables.

In accordance with the RECIST criteria (see [5]) we have evaluated the tumor area evolution from the CT scans of the two patients, shown in Fig 2, measured as the product of the largest and smallest diameters of the tumor observed in each image. As a result, for each patient we have a data set

$$\begin{array}{c} A_{i}^{j},\;\text{for}\;i=1,\ldots ,N^{j},\;\text{for}\;j=1,2, \end{array}$$

where the $A_{i}^{j}$ denotes the tumor area evaluated for patient *j* at time $t_{i}^{j}$ for *i* = 1, …, *N*^*j*^, and *N*^*j*^ is the total number of CT scans available for the patient *j*.

For the *direct problem* associated to our general model, given a parameter vector $\theta \in R^{8}$, the unique solution *U*(*t*, *θ*) of the mathematical model given by Eqs (11) and (12) must be found. This solution is required to solve the *inverse problem of parameter estimation*, *i.e*. given the tumor area evolution observed from the CT scans of the two patients, namely ${\left\{A_{i}^{j}\right\}}_{i=1}^{N^{j}}$ for *j* = 1, 2, a parameter vector $\theta^{j}\in R^{8}$ must be identified such that the mathematical model given by Eqs (11) and (12) fits data in the sense of the least squares.

In the proposed models summarized in Table 2, *P*(*t*) = (*P*_1_ + *P*_2_ + *P*_3_)(*t*) represents the total tumor volume at time *t*, which is proportional to the tumor area A(t). Indeed, by assuming that the tumor is ellipsoid-shaped, then its volume V(t) is given by V(t)=(4πc/3)A(t), where *c* is set as *c* = 3/4*π* mm. Only this partial combination of model variables, referred from now on as *model’s observation function*, is observed. In order to identify the model’s parameters a vector $\theta^{j}\in R^{8}$ must be found such that the sum of squares

$$\begin{array}{ccc} S(\theta^{j}) & = & \sum_{i=1}^{N^{j}}{\left(A_{i}^{j}-P\left(t_{i}^{j},\theta^{j}\right)\right)}^{2} \end{array}$$ (14)

is minimized with respect to the data set ${\left\{A_{i}^{j}\right\}}_{i=1}^{N^{j}}$, for each model’s observation function $P(t_{i}^{j},\theta^{j})$ *j* = 1, 2. The objective function in Eq (14) is appropriate under the assumption of constant variance measurements, *i.e*. the measurement error is considered to be a random variable with constant variance (see S1 Appendix).

Under the assumption of non-constant variance measurements, *i.e*. the measurement error is considered to be a random variable with non-constant variance, in order to identify the model’s parameters a vector $\theta^{j}\in R^{8}$ must be found such that the sum of squares

$$\begin{array}{ccc} S(\theta^{j}) & = & \sum_{i=1}^{N^{j}}{\left(\frac{A_{i}^{j}-P\left(t_{i}^{j},\theta^{j}\right)}{A_{i}^{j}}\right)}^{2}\;. \end{array}$$ (15)

is minimized (see S1 Appendix).

As discussed before, the proposed mathematical models may not be uniquely identifiable, since with different sets of parameters any of them is capable of reproducing a given initial tumor behavior, while representing very different scenarios in the long-term (see Fig 1 right). We believe this issue would be solved by considering information regarding the specific proportion of sensitive and resistant to treatment tumor cell populations for each patient. As a result, the objective function *S*(*θ*^*j*^) could not have a unique global minimum but several local minima. This feature is typically observed in inverse problems that are ill-posed, meaning a small perturbation on the observed data can lead to a big perturbation in the obtained solution. In particular, it has been observed that parameter identification problems in biological systems are generally ill-posed (see [10, 11] and references therein). To overcome this difficulty, we introduce a regularization term in the optimization problem, *i.e*. instead of minimizing *S*(*θ*^*j*^), we minimize

$$\begin{array}{c} \min_{\theta^{j}\in R^{8}}S\left(\theta^{j}\right)+\kappa {∥\theta^{j}∥}_{2}^{2}\;, \end{array}$$ (16)

where *κ* > 0 is the regularization parameter. We solve the optimization problem in Eq (16) for both sums of squares in Eqs (14) and (15) for several values of *κ* small enough, obtaining the best results for *κ* = 0.001.

The optimal solution of the regularized sum of squares in Eq (16), ${\hat{\theta }}^{j}$ for *j* = 1, 2, is called *nonlinear least squares estimator* (*nonlinear LSE*). In order to minimize the objective function given by Eq (16), we utilize the *Nelder-Mead simplex algorithm* implemented in Matlab^®^ under the subroutine *fminsearch*. Details on the numerical method, as well as on its implementation are discussed in the S2 Appendix.

Once the nonlinear LSE ${\hat{\theta }}^{j}$ has been found for *j* = 1, 2 and each of the five proposed models summarized in Table 2, models are compared based on their fit performance to clinical data by computing goodness of fit criteria. In addition, the two parameter vectors obtained for the objective functions described in Eqs (14) and (15) are compared in terms of their residual plots to identify the most suitable one for obtaining the best parameter estimation results, as explained in the S1 Appendix.

#### Assessing goodness of fit

Statistical methods, in the context of non-linear least squares regression, are utilized to quantify the reliability of the parameters estimated. They are also utilized to evaluate the robustness of the proposed models for quantitatively describing drug resistance for each patient. Once the nonlinear LSE is found for *j* = 1, 2 and each of the five proposed models summarized in Table 2, goodness of fit criteria can be computed to evaluate to what extent these models fit the empirical data. The goodness of fit criteria computed in this work are the following (see [12]; see also pp. 229 in [13]):

$$\begin{array}{c} {\left({\tilde{\sigma }}^{j}\right)}^{2}=\frac{1}{N^{j}}S\left({\hat{\theta }}^{j}\right) \end{array}$$ (17)

is the variance of the residuals. Similarly,

$$\begin{array}{c} {\left({\hat{\sigma }}^{j}\right)}^{2}=\frac{1}{N^{j}-m}S\left({\hat{\theta }}^{j}\right), \end{array}$$ (18)

is the unbiased variance, where *m* is the number of parameters estimated. Moreover,

$$\begin{array}{ccc} {\hat{\sigma }}^{j} & = & \sqrt{{\left({\hat{\sigma }}^{j}\right)}^{2}}=\sqrt{\frac{1}{N^{j}-m}S\left({\hat{\theta }}^{j}\right)}\;, \end{array}$$ (19)

$$\begin{array}{ccc} R^{2,j} & = & 1-\frac{{\left(e^{j}\right)}^{t}e^{j}}{\sum_{i=1}^{N^{j}}{\left(A_{i}^{j}-{\bar{A}}^{j}\right)}^{2}}\;, \end{array}$$ (20)

are the *Root Mean Squared Error (RMSE)* ${\hat{\sigma }}^{j}$, and the *coefficient of determination* *R*^2,*j*^, respectively, where ${\bar{A}}^{j}$ in Eq (20) denotes the mean of the observations ${\left(A_{i}^{j}\right)}_{i=1}^{N^{j}}$, and the vector $e^{j}\in R^{N^{j}}$ stands for the *absolute residuals* defined by

$$\begin{array}{c} e_{i}^{j}=A_{i}^{j}-P\left(t_{i}^{j},{\hat{\theta }}^{j}\right)\;i=1,\ldots ,N^{j}. \end{array}$$ (21)

Finally, in order to verify whether the measuring errors are normally distributed or not, the one-sample Kolmogorov-Smirnov (K-S) test [14] is applied. This statistical test is implemented in Matlab^®^ through the *kstest* subroutine. Verifying the normality assumption is required in order to accurately compute the standard errors associated with the nonlinear LSE.

## Results and discussion

Numerical results obtained by applying the methodology described in the Practical identification approach section are presented and discussed. The details on the numerical method and its implementation in order to solve the parameter estimation problem are provided in the S2 Appendix. The goodness of fit to data, as well as the validity of the statistical assumptions for obtaining the best parameter estimation results for each of the proposed models are also assessed according to the methods described in theAssessing goodness of fit subsection and in the S1 Appendix, respectively.

### Parameter estimation results

The estimated parameters for our proposed models, obtained as described in the Parameter estimation problem section, are presented and discussed. Results obtained for each data set are reported separately. The fit for each objective function, based on the sum of squares given by Eqs (14) and (15), the obtained optimal parameters, as well as the corresponding values for both sum of squares for each of the proposed models are shown. Finally, the plot for the two best models based on the obtained optimal sum of squares under each statistical assumption is presented.

#### Fit to Patient’s 1 data under a constant variance assumption

**Fig 3:**
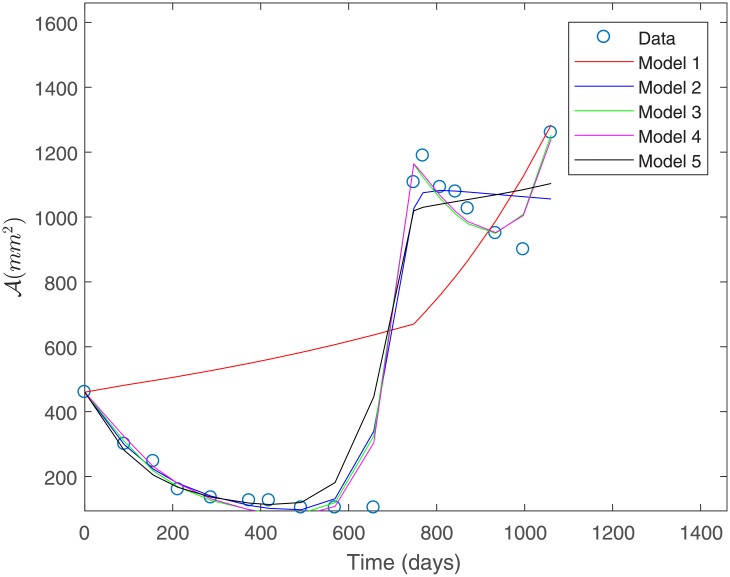
Fit to Patient’s 1 data under a constant variance assumption.

The fit to the data for Patient 1 for all the proposed models is shown in Fig 3. This fit was obtained under a constant variance data assumption as discerned by the sum of squares defined in Eq (14).

From Fig 3 we observe that Model 1 is the least suitable for describing Patient’s 1 data, whereas Models 3 and 4 provide a good fit. Both of these models consider a basal vasculature and a direct effect of the second-line treatment on the sensitive populations *P*_1_ and *P*_2_. This suggests that these factors might be physiologically relevant in capturing the response to treatment observed for Patient 1. Table 3 shows the estimated parameters for each of the proposed models in Fig 3. We observe that parameter values are all positive and within biologically meaningful ranges.

**Table 3:** Estimated model parameters for Patient’s 1 data under a constant variance assumption.

Model №	${\hat{\mu }}_{\text{MAX}}$	${\hat{\delta }}_{\text{MAX}}$	${\hat{M}}_{\text{hyp}}$	${\hat{\delta }}_{1}$	${\hat{\delta }}_{2}$	$\hat{\gamma }$	$\hat{\beta }$	$\hat{\nu }\;\text{or}\;\hat{\zeta }$
1	0.2430	0.0015	0.0870	0.0359	0.4221	0.0004	0.0014	0.6557
2	0.2759	0.0023	0.0900	0.0128	0.4952	0.0003	0.0029	0.3314
3	0.7802	0.5609	0.0222	0.0148	0.0300	0.0003	0.0105	0.3864
4	0.4854	0.0081	0.0216	0.0142	0.3202	0.0001	0.0081	0.8183
5	0.5263	0.0215	0.1446	0.0557	0.4075	0.0003	0.0049	0.1260

Table 4 shows the computed sum of squares of absolute errors in Eq (14), as well as the sum of squares of relative errors in Eq (15) associated to the fit of each model to Patient’s 1 data.

**Table 4:** Sums of squares of absolute and relative errors for Patient’s 1 data under a constant variance assumption.

Sums of squares		
Model №	Absolute	Relative
1	1.4799e+3	10.4424
2	403.6207	2.3188
3	282.9373	2.1798
4	260.6881	1.9857
5	485.0905	3.3926

**Fig 4:**
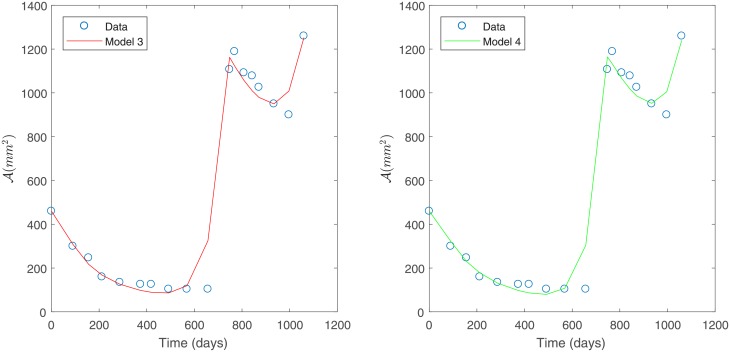
Best models for Patient’s 1 data. Predicted tumor area for Patient’s 1 data under a constant variance assumption. Model 3 (left) and Model 4 (right).

Under constant variance data assumption, the best fit is obtained for Models 3 and 4 as they have the smallest sum of squares of absolute errors, as shown in Table 4; see Fig 4. This corroborates the observations made from Fig 3.

#### Fit to Patient’s 1 data under a non-constant variance assumption

**Fig 5:**
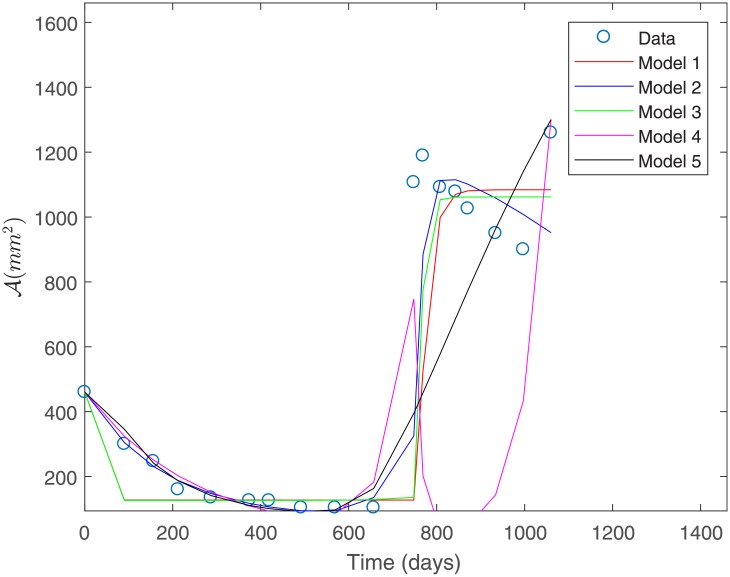
Fit to Patient’s 1 data under a non-constant variance assumption.

Results under a non-constant variance data assumption as discerned by the sum of squares defined in Eq (15) are presented below. Fig 5 shows the estimated fits for all the proposed models. Table 5 presents the estimated parameters under non-constant variance assumption for each of the proposed models.

**Table 5:** Estimated model parameters for Patient’s 1 data under a non-constant variance assumption.

Model №	${\hat{\mu }}_{\text{MAX}}$	${\hat{\delta }}_{\text{MAX}}$	${\hat{M}}_{\text{hyp}}$	${\hat{\delta }}_{1}$	${\hat{\delta }}_{2}$	$\hat{\gamma }$	$\hat{\beta }$	$\hat{\nu }\;\text{or}\;\hat{\zeta }$
1	0.7845	0.4636	0.0109	0.0287	0.1164	0.0005	0.0009	-1.9507
2	0.1697	0.0060	0.0927	0.5224	0.4016	0.0005	0.0032	0.2338
3	0.4753	0.7390	0.0135	-0.0258	0.1678	0.0023	0.0008	-1.2269
4	0.0294	-0.0129	0.0212	0.0809	0.1026	0.0003	0.0141	0.1931
5	0.4113	0.0373	0.2442	0.0479	0.2805	0.0001	0.0015	0.0195

Results in Fig 5 show a deficient fit for all models to Patient’s 1 data. In addition, negative parameter values in Table 5 indicate a defective overall fit for Models 1, 3 and 4 due to the fact that the non-constant variance data assumption is not suitable for Patient’s 1 data. This is corroborated in the Modeling capabilities. subsection, through residual plots as explained in the S1 Appendix.

Finally, Table 6 presents the sum of squares of relative errors in Eq (15), as well as the sum of squares of absolute errors in Eq (14) associated to each model obtained under a non-constant variance data assumption.

**Table 6:** Sums of squares of relative and absolute errors for Patient’s 1 data under a non-constant variance assumption.

Sums of squares		
Model №	Relative	Absolute
1	1.3953	1.2408e+3
2	0.9137	909.3604
3	1.3191	1.1144e+3
4	2.3342	2.2730e+3
5	1.3150	1.2606e+3

**Fig 6:**
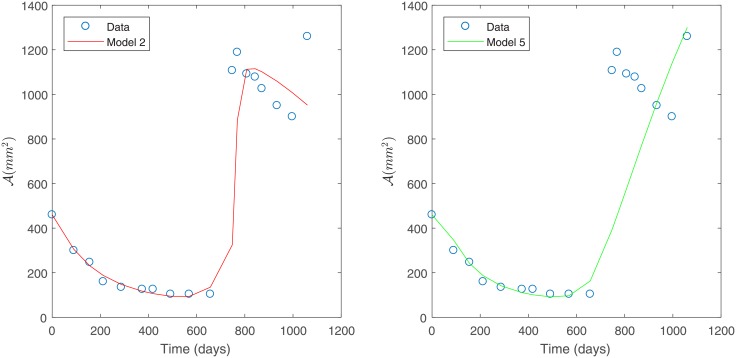
Best models for Patient’s 1 data. Predicted tumor area for Patient’s 1 data under a non-constant variance assumption, Model 2 (left) and Model 5 (right).

The best two models (Models 2 and 5) were selected based on the smallest sum of squares of relative errors, according to Table 6. These models are shown in Fig 6.

Unlike what was observed in Fig 4, based on the results shown in Fig 6 even the best models obtained are not as suitable for fitting Patient’s 1 data independent of the model assumptions considered (detailed in Table 2). This is due to the fact that a non-constant variance data assumption is not suitable for Patient’s 1 data as discussed in the Modeling capabilities. subsection.

#### Fit to Patient’s 2 data under a constant variance assumption

**Fig 7:**
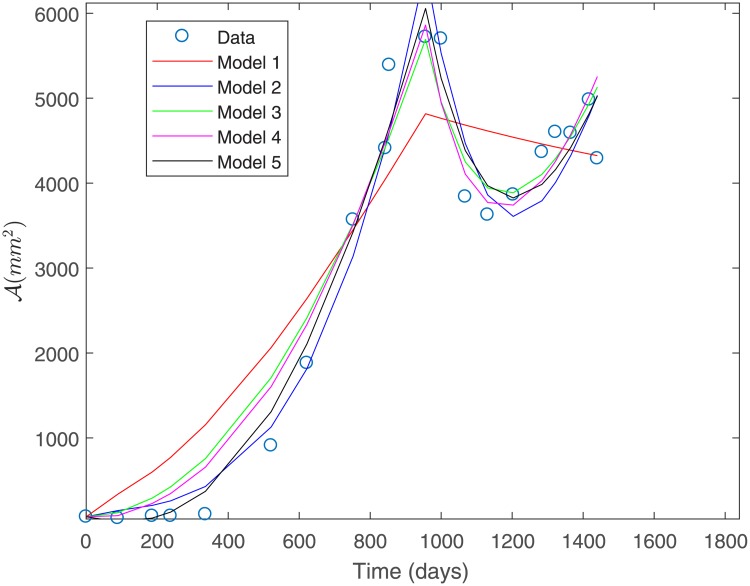
Fit to Patient’s 2 data under a constant variance assumption.

The fit to the data for Patient 2 for all the proposed models is shown in Fig 7. This fit was obtained under a constant variance data assumption as discerned by the sum of squares defined in Eq (15).

From Fig 7 we can observe that Model 1 is the least suitable for fitting Patient’s 2 data, while Models 3, 4 and 5 provide a good fit. Table 7 shows the estimated parameters for each of the proposed models shown in Fig 7.

**Table 7:** Estimated model parameters for Patient’s 2 data under a constant variance assumption.

Model №	${\hat{\mu }}_{\text{MAX}}$	${\hat{\delta }}_{\text{MAX}}$	${\hat{M}}_{\text{hyp}}$	${\hat{\delta }}_{1}$	${\hat{\delta }}_{2}$	$\hat{\gamma }$	$\hat{\beta }$	$\hat{\nu }\;\text{or}\;\hat{\zeta }$
1	0.8712	0.4995	0.0455	0.0912	0.0564	0.0003	0.0093	0.6028
2	0.9668	0.0026	0.0604	0.1610	0.5992	0.0001	0.0053	0.3942
3	0.4925	0.0698	0.1699	0.0073	0.1972	0.0002	0.0020	0.2521
4	0.2986	0.0886	0.1694	0.0081	0.1235	0.0002	0.0058	0.4640
5	0.4131	0.0038	0.1505	0.0217	0.7332	0.0002	0.0024	0.7010

As for Patient’s 1 data, we can observe from Table 7 that parameter values for Patient’s 2 data are all positive and within biologically meaningful ranges. Table 8 shows the computed sum of squares of absolute errors defined in Eq (14), as well as the sum of squares of relative errors associated to the fit of each model to Patient’s 2 data.

**Table 8:** Sums of squares of absolute and relative errors for Patient’s 2 data under a constant variance assumption.

Sums of squares		
Model №	Absolute	Relative
1	3.1508e+3	15.8906
2	1.8806e+3	4.5527
3	1.9865e+3	8.3845
4	1.8899e+3	6.7809
5	1.5882e+3	2.9447

**Fig 8:**
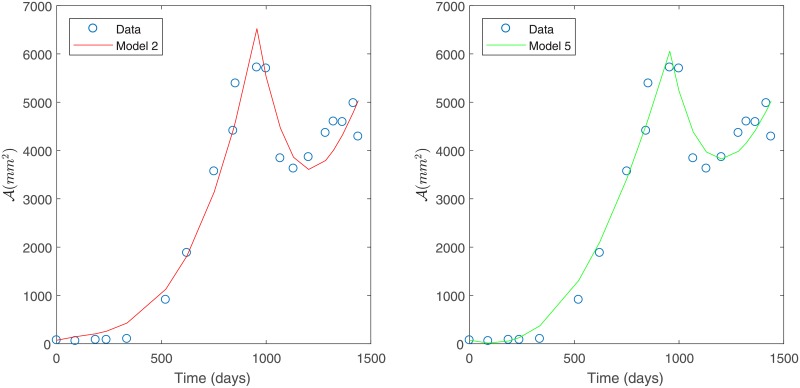
Best models for Patient’s 2 data. Predicted tumor area for Patient’s 2 data under a constant variance assumption. Model 2 (left) and Model 5 (right).

Under constant variance data assumption, the best two models (Models 2 and 5) were selected based on the smallest sum of squares of absolute errors according to Table 8. These models are shown in Fig 8. However, we remark that Model 4 has a sum of squares of absolute errors only slightly greater than Model 2.

#### Fit to Patient’s 2 data under a non-constant variance assumption

**Fig 9:**
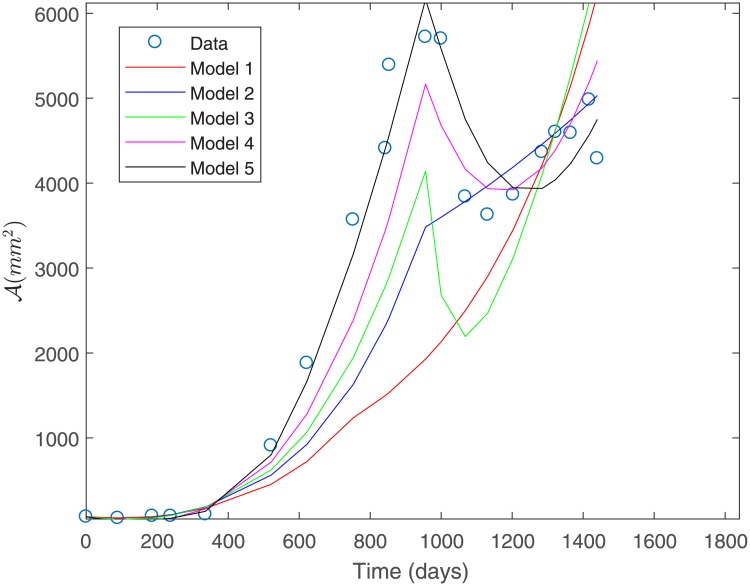
Fit to Patient’s 2 data under a non-constant variance assumption.

Finally, the fit to Patient’s 2 data for each of the proposed models under a non-constant variance assumption, as discerned by the sum of squares defined in Eq (15), is shown in Fig 9.

Table 9 presents the estimated parameters for each of the proposed models in Fig 9. Despite the fact that data in Table 9 shows all parameter values to be possitive and within biologically meaningful ranges, Fig 9 shows a deficient fit to data for all models, except for Models 4 and 5.

**Table 9:** Estimated model parameters for Patient’s 2 data under a non-constant variance assumption.

Model №	${\hat{\mu }}_{\text{MAX}}$	${\hat{\delta }}_{\text{MAX}}$	${\hat{M}}_{\text{hyp}}$	${\hat{\delta }}_{1}$	${\hat{\delta }}_{2}$	$\hat{\gamma }$	$\hat{\beta }$	$\hat{\nu }\;\text{or}\;\hat{\zeta }$
1	0.1120	0.0011	0.1402	0.0636	0.3474	0.0003	0.0007	0.3969
2	0.0768	0.0069	0.2598	0.0806	0.2313	0.0003	0.0032	0.1909
3	0.0162	0.0200	0.0203	0.0213	0.0001	0.0006	0.0002	0.0284
4	0.8953	0.0012	0.1472	0.0043	0.8260	0.0001	0.0052	1.0746
5	1.0419	0.0036	0.2306	0.0256	0.7499	0.0000	0.0003	0.9439

In Table 10 results for the sum of squares of the relative errors defined in Eq (15), as well as their associated sum of squares of absolute errors for the fit of each model to Patient’s 2 data.

**Table 10:** Sums of squares of relative and absolute errors for Patient’s 2 data under a non-constant variance assumption.

Sums of squares		
Model №	Relative	Absolute
1	1.9486	8.0488e+3
2	1.5855	5.3104e+3
3	1.6581	5.9391e+3
4	1.4813	2.9960e+3
5	1.1471	1.9239e+3

**Fig 10:**
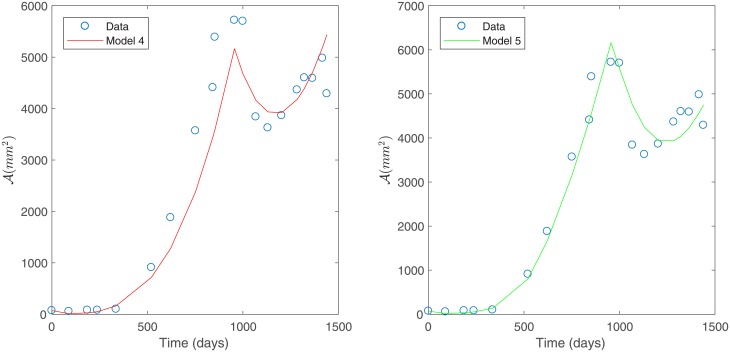
Best models for Patient’s 2 data. Predicted tumor area for Patient’s data under a non-constant variance assumption. Model 4 (left) and Model 5 (right).

Under a non-constant variance data assumption, the best two models (Models 4 and 5) were selected based on the smallest sum of squares of relative errors as given in Table 10. This model selection is in agreement with what was observed in Fig 9. Selected models are shown in Fig 10.

Based on the relative errors in Table 10, Model 5 is slightly better than Model 4. Both of these models consider a direct effect of treatment on the sensitive tumor cells and a nutrient consumption rate that is dependent on cell growth (*β*(*μ*)). This suggest that environmental factors related to nutrient availability are more relevant in the case of Patient 2. The main difference between these two variants is that Model 4 considers a basal vasculature while Model 5 does not, indicating that accounting for a basal vasculature is not significant for this data set (see Table 2 for a summary of the model variants considered). This is consistent with what is observed in Fig 10.

### Comparison of the proposed models

The proposed models are compared in a quantitative manner, by assessing the goodness of fit to data as described in the Assessing goodness of fit. At the same time, the statistical assumptions behind of the sums of squares defined by Eqs (14) and (15) are analyzed, by plotting the residuals for Models 1 to 5 to each patient’s data, and for both absolute and relative error specifications. See S1 Appendix for a detailed description. The validation of one of these assumptions is required in order to achieve appropriate parameter estimation results. The correct assumption is validated before comparing fit performance for each model. This is performed after carrying out a parameter estimation, since the residual plots associated to the nonlinear LSE are required for this task.

Figures are placed at the end of the present subsection for improved readability.

#### Comparison for Patient’s 1 data

**Fig 11:**
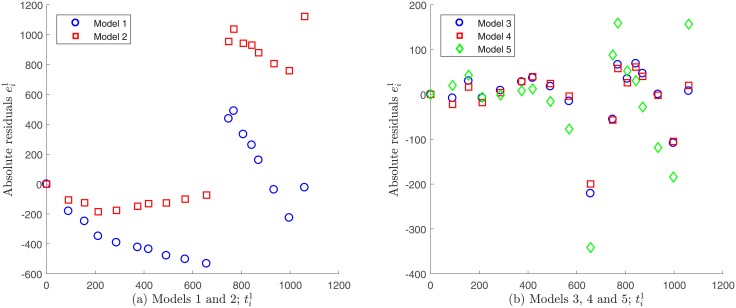
Absolute residuals vs. time for Patient’s 1 data under constant variance assumption. A random pattern is observed for Models 3, 4 and 5, whereas a certain tendency is observed for models 1 and 2.

**Fig 12:**
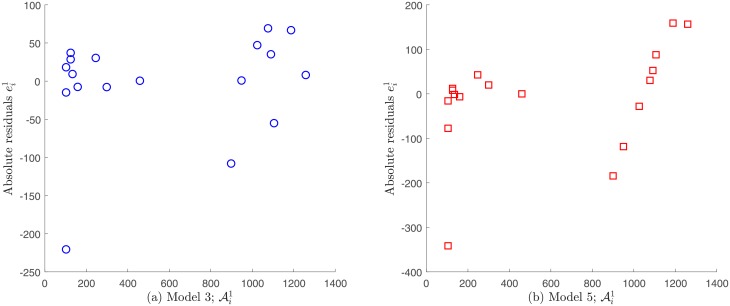
Absolute residuals vs. observations for Patient’s 1 data under a constant variance assumption. A random pattern is observed for Models 3 and 4 supporting the assumption of constant variance generated data, whereas a certain tendency is observed for Model 5. Note that residuals for Model 4 are not shown since they are very similar to ones for Model 3 (see Fig 11).

Models 1 and 2 do not verify the statistical assumption on error independence $\varepsilon_{i}^{1}$, and therefore accurate results for the related parameter estimation may not be expected. This finding is supported by the plot of the absolute residuals $e_{i}^{1}$ given by Eq (21) vs. time, which is shown in Fig 11. A random pattern would be a strong support for the validity of the independence assumption, but this not observed in the case of Models 1 and 2; see S1 Appendix. In contrast, Models 3, 4 and 5 seem to fulfill this requirement as shown in Fig 11. Moreover, by plotting the absolute residuals $e_{i}^{1}$ vs. observations $A_{i}^{1}$, the assumption of constant variance data as discerned by Eq (14) seems to be reasonable only for Models 3 and 4, since a random pattern was also obtained for them, whereas a certain tendency was observed for Model 5; see Fig 12. This indicates that the variance does not depend on the observations $A_{i}^{1}$ for Models 3 and 4, validating constant variance assumption as discerned by Eq (14). Note that under this assumption, we have already obtained that these models best fitted to Patient’s 1 data (see Fig 4). Next we evaluate the goodness of fit criteria for these models only, in order to quantitatively compare them. Table 11 shows the values for the goodness of fit criteria described in the Assessing goodness of fit subsection for Models 3 and 4.

**Table 11:** Statistics for the goodness of fit criteria to Patient’s 1 data under constant variance assumption.

Model №	${\tilde{\sigma }}^{2}$	RMSE	R^2^
3	4.4474*e* + 3	89.4726	0.9785
4	3.7755*e* + 3	82.4368	0.9818

Results in Table 11 indicate that a better fit is achieved by Model 4 followed by Model 3. In consequence, once the statistical assumption on measuring errors is validated, we can confirm that Model 4 is the best fit to Patient’s 1 data.

On the other hand, standard errors computation, which are necessary to quantitatively evaluate the uncertainty of the estimated parameters for Models 3 and 4, is valid only if residuals are normally distributed or if data numbers are sufficiently large; see [12]. In this regard, to verify the normality assumption we compute *p*-values for the one-sample Kolmogorov-Smirnov test [14], shown in Table 12.

**Table 12:** One-sample K-S test for normality of residuals for Patient’s 1 data.

Model №	p-value
3	9.3543e-6
4	9.3793e-6

Results in Table 12 show that the normality assumption is not valid. In addition, the number of data points for each patient *j* = 1, 2 is relatively small, therefore standard errors cannot be accurately computed. In consequence, standard errors were excluded from our analysis, and the uncertainty of parameter estimation for Models 3 and 4 was considered to be assessed through validation of the statistical assumption satisfied for the measuring errors, as a qualitative way to ensure the accuracy of parameter estimation.

**Fig 13:**
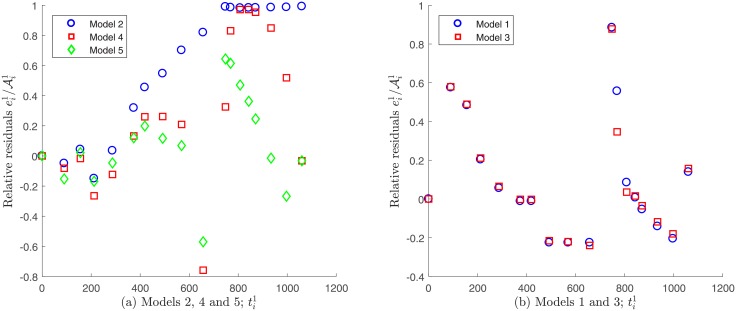
Relative residuals vs. time for Patient’s 1 data, assuming a non-constant variance. A certain tendency is observed for all the proposed models.

Regarding the non-constant variance assumption as discerned in Eq (15), it can be observed in Fig 13 that none of the models seem to fulfill the error independence assumption, since the relative residuals $e_{i}^{1}/A_{i}^{1}$ vs. time $t_{i}^{1}$ appear to have a certain pattern. In consequence, in this case it would not adequate to validate the non-variance data assumption as discerned by Eq (15) in order to solve the parameter estimation problem.

In summary, the best models for fitting Patient’s 1 data are Models 4 and 3 under constant variance data assumption. This finding suggests that the physiological mechanism describing therapy failure, due to drug resistance for Patient 1, should consider a basal vasculature and that the second-line treatment would have a direct effect on the sensitive tumor cells (see Table 2 for a summary of the models proposed). In addition, as shown in Table 11, Model 4 that considers nutrient consumption rate to be proportional to the normalized growth rate of tumor cells, is only slightly better than Model 3 with a constant nutrient consumption rate. Hence, the dependence of the nutrient consumption rate on the normalized growth rate appears not to be as significant for this data set. This is consistent with what is observed in Fig 4.

#### Comparison for Patient’s 2 data

**Fig 14:**
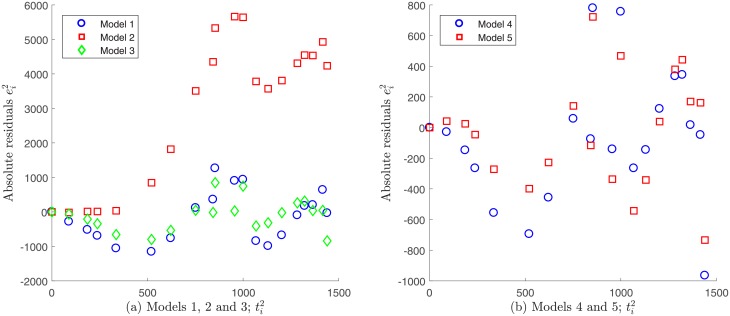
Absolute residuals vs. time for Patient’s 2 data under a constant variance assumption. A certain tendency is observed for Models 1, 2 and 3, whereas a random pattern is observed for Models 4 and 5.

**Fig 15:**
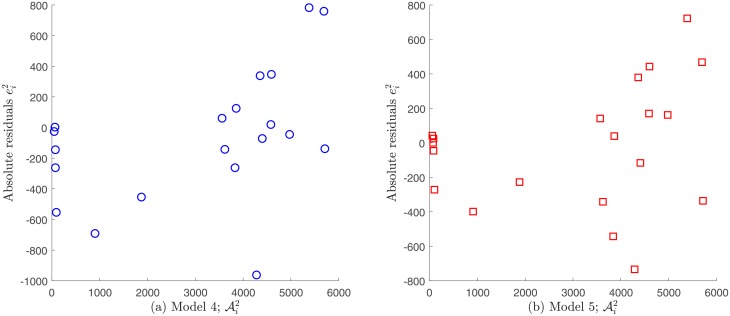
Absolute residuals vs. observations for Patient’s 2 data under a constant variance assumption. A random pattern is observed for Models 4 and 5 supporting the assumption of constant variance generated data.

Following the same analysis described above for the case of Patient’s 1 data, we do not consider Models 1, 2 and 3 for the case of Patient’s 2 data, as they do not exhibit independence neither the errors vs. time nor on errors vs. observations. Models 4 and 5 fulfill these assumptions and were analyzed further; see Figs 14 and 15. Table 13 shows goodness of fit criteria values described in the Assessing goodness of fit subsection.

**Table 13:** Statistics for the goodness of fit to Patient’s 2 data under constant a variance assumption.

Model №	${\tilde{\sigma }}^{2}$	RMSE	R^2^
4	1.7843e+05	5.4533e+02	0.95811
5	1.2611e+05	4.5846e+02	0.97039

Data in Table 13 show that the best fit is achieved by Model 5 followed by Model 4. The accuracy of parameter estimation for Models 4 and 5 is considered qualitatively assessed through validation of the statistical assumption satisfied for the measuring errors.

**Fig 16:**
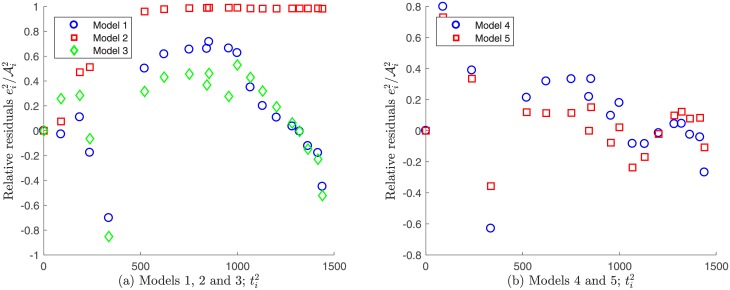
Relative residuals vs. time for Patient’s 2 data under a non-constant variance assumption. A certain tendency is observed for Models 1, 2 and 3, whereas a random pattern is observed for Models 4 and 5.

Regarding the non-constant variance assumption in Eq (15), it can be observed from Fig 16 that the only possible candidates to fulfill the error independence assumption are models 4 and 5. However, since it was already verified that data generated by these models have constant variance measuring errors, it is impossible that they satisfy the opposite assumption. In consequence, for Patient’s 2 data the correct statistical assumption on measuring errors is also to consider constant variance generated data.

In summary, the best models for fitting Patient’s 2 data are Models 5 and 4 under constant variance data assumption. This finding suggests that the physiological mechanism describing therapy failure due to drug resistance for Patient 2, should consider that the second-line treatment would have a direct effect on the sensitive tumor cells and that the nutrient consumption rate should be considered as proportional to the normalized growth rate of tumor cells (see Table 2 for a summary of the proposed model variants). On the other hand, results in Table 13 show that Model 5 is only slightly better than Model 4, which suggests that considering a basal vasculature is not as relevant for this data set.

## Summary and conclusions

In this work we have implemented a practical identifiability approach aiming to quantitatively describe therapy failure due to drug resistance in GIST metastasis to the liver, using patient-specific mathematical models. Specifically, we proposed a general modeling framework for metastatic tumor cell growth and therapy failure. Five model variants, which represent different relevant physiological mechanisms, were proposed. Parameters for these models represent biologically meaningful quantities regarding cell growth and death, among others, and what is very important, variables quantitatively describe therapy failure.

Parameter estimation was carried out for these five model variants in order to assess their fit performance, using observations for tumor area obtained from two patients, which are representative of the two possible outcomes observed clinically for GIST metastasis in response to treatment under a drug resistance scenario. In addition, to improve parameter estimation results the validity of the statistical assumptions on errors (absolute and proportional to experimental data) was evaluated.

Our results indicate that the constant variance data assumption is the most suitable for the experimental data available. Under this assumption, Model 4 followed by Model 3 were the best fit to Patient’s 1 data, whereas Model 5 followed by Model 4 were the best fit for Patient’s 2 data. We believe that Models 3 and 4 are the best fit to describe 85% of the patients responses, represented by patient’s 1 data set, and that Models 5 and 4 are the best fit for describing the remaining 15% of cases. From a physiological point of view, we can infer that the second-line treatment acts on the sensitive tumor cells for both patient’s cases, since models 3, 4 and 5 share this mechanism. On the other hand, the main differences between the best models for both patients would be associated to the existence of a basal vasculature and to the dependence of nutrient consumption on the normalized growth rate of tumor cells. Since for Patient’s 2 data, Models 4 and 5 have similar goodness of fit statistics (see Table 13) under an absolute error assumption, we consider Model 4 to be the best consensus fit to both data sets. Under this consensus, we can consider that tumor evolution for both patient’s cases would be associated to the presence of a basal vasculature and a nutrient consumption rate that is dependent on the normalized tumor growth rate.

In conclusion, we have successfully obtained phenomenological models that are able to capture the therapy failure responses that has been clinically observed in patients with GIST metastasis to the liver showing drug resistance. This is the first work that reports capturing therapy failure based on clinical images in a patient-specific manner, by using a mathematical model. The obtained models allow us to quantitatively describe therapy failure to treatment of GIST metastasis to the liver by using available observations, which could contribute to the design of new therapeutic strategies that minimize drug resistance. Additional studies need to be conducted in order to provide enough information to elucidate the underlying mechanisms of resistance, before developing mathematical models that consider additional mechanistic details and that may explain this phenomenon in a more accurate way. In particular, an experimental framework that would estimate the actual proportion of sensitive/resistant to treatments tumor cells could help to obtain a more accurate quantitative description of the involved physiological mechanism behind drug resistance.

The methodology presented in this work could also be applied in the context of therapy failure due to drug resistance to other biological systems with empirical observations, where a phenomenologically based mathematical model can be proposed, and where parameter identification is likely to be a problem due to a scarce availability of data.

## Supporting information

S1 Appendix Statistical assumptions on the measuring errors. (PDF)

S2 Appendix Numerical method. (PDF)

S1 Table Patient’s 1 data. (XLSX)

S2 Table Patient’s 2 data. (XLSX)
