# Supplementary material for: Parameter estimation and mathematical modeling for the quantitative description of therapy failure due to drug resistance in gastrointestinal stromal tumor metastasis to the liver
Source: PLoS One. 2019 May 30;14(5):e0217332. doi: 10.1371/journal.pone.0217332 (PMC6542538; doi:10.1371/journal.pone.0217332)
Supplement: S1 Appendix — (PDF) [file pone.0217332.s001.pdf]

S1 Appendix: Statistical assumptions on the measuring errors

In minimizing the sum of squares defined by

$$S(\theta^j) \;=\; \sum_{i=1}^{N^j} \left(\mathcal{A}_i^j - P(t_i^j, \theta^j) \right)^2 \tag{1}$$

data are generated assuming constant variance measurements, *i.e.* the errors ε_i^j made in measuring data $\mathcal{A}_i^j$ do not depend on them. Alternatively, in minimizing the sum of squares defined by

$$S(\theta^j) \;=\; \sum_{i=1}^{N^j} \left(\frac{\mathcal{A}_i^j - P(t_i^j, \theta^j)}{\mathcal{A}_i^j} \right)^2 . \tag{2}$$

data are generated assuming non-constant variance measurements, *i.e.* the errors ε_i^j made in measuring data $\mathcal{A}_i^j$ are assumed to be proportional to them (see [1, 2]). More precisely, to identify the model's parameters we assume that there is an underlying probability distribution or data generating process (DGP) for the observable quantities $\mathcal{A}_i^j$ and a true parameter vector, θ_0^j, which is a characteristic of that DGP. In other words, the observable quantities $\mathcal{A}_i^j$ are randomly generated by the nonlinear regression models

$$\mathcal{A}_i^j \;=\; P(t_i^j, \theta_0^j) + \varepsilon_i^j \quad \text{or,} \tag{3}$$

$$\mathcal{A}_i^j \;=\; P(t_i^j, \theta_0^j) + \mathcal{A}_i^j \varepsilon_i^j \, . \tag{4}$$

In Eq (3) data are generated assuming an absolute error model, *i.e.* the errors ε_i^j do not depend on the observed quantities $\mathcal{A}_i^j$. Alternatively, in Eq (4) data are generated assuming a proportional error model, *i.e.* the errors ε_i^j are assumed proportional to the size of the observed quantities $\mathcal{A}_i^j$ (see [1, 2]). In both cases, the errors ε_i^j are assumed to be random variables following a well-defined probability distribution. The main assumption behind is that the DGP produces a sample of *independent, identically distributed (i.i.d.)* random variables ε_i^j with mean zero and variance $(\sigma^j)^2$ conditioned on the model observation function $P(t_i^j, \theta_0^j)$. The nonlinear regression model is said to be *semiparametric*, since no assumptions regarding any particular distribution for ε_i^j are made. For a detailed description of the statistical assumptions we refer to [1] and pp. 223 in [3]. Both measuring errors assumptions were investigated in order to obtain reliable results for parameter estimation. We emphasize that not choosing the good assumption on the measuring errors can lead to incorrect conclusions. This is explained in the rest of this appendix based on reference [1].

The tool required in order to validate the statistical assumptions on the measuring errors are the *residual plots*. In the case of an absolute error model given by Eq (3), we utilize the absolute residuals $e_i^j = \mathcal{A}_i^j - P(t_i^j, \hat{\theta}^j) \quad i = 1, \ldots, N^j$, to test whether the data set $\{\mathcal{A}_i^j\}_{i=1}^{N^j}$ is i.i.d. and has the assumed variance structure. If the data set has constant variance error then from Eq (3), one has that $\varepsilon_i^j = \mathcal{A}_i^j - P(t_i^j, \theta_0^j)$. Since the errors $\{\varepsilon_i^j\}_{i=1}^{N^j}$ are assumed to be i.i.d., a plot of the residuals $e_i^j = \mathcal{A}_i^j - P(t_i^j, \hat{\theta}^j)$ vs. t_i^j for $i = 1, \ldots, N^j$ should exhibit a random pattern for each $j = 1, 2$. Moreover, the errors in the constant variance case do not depend on $\mathcal{A}_i^j$, and so a plot of the residuals $e_i^j = \mathcal{A}_i^j - P(t_i^j, \hat{\theta}^j)$ vs. $\mathcal{A}_i^j$ for $i = 1, \ldots, N^j$ should also exhibit a random pattern for each $j = 1, 2$. Therefore, if the errors $\{\varepsilon_i^j\}_{i=1}^{N^j}$ have constant variance then a plot of the residuals $e_i^j = \mathcal{A}_i^j - P(t_i^j, \hat{\theta}^j)$ vs. t_i^j and vs. $\mathcal{A}_i^j$ should both show a random pattern. If not, the validity of the constant variance assumption is uncertain. See the reference [1] for details.

In the case of a proportional error model given by Eq (4), the residuals $e_i^j = \mathcal{A}_i^j - P(t_i^j, \hat{\theta}^j) \approx \mathcal{A}_i^j - P(t_i^j, \theta_0^j) = \mathcal{A}_i^j \varepsilon_i^j$ for $i = 1, \ldots, N^j$ would depend on the observed quantities $\{\mathcal{A}_i^j\}_{i=1}^{N^j}$, and then one should expect that a plot of the residuals $e_i^j = \mathcal{A}_i^j - P(t_i^j, \hat{\theta}^j)$ vs. t_i^j for $i = 1, \ldots, N^j$ should exhibit some type of pattern. Moreover, the residuals e_i^j depend on $\mathcal{A}_i^j$ in the non-constant variance case, and so as $\mathcal{A}_i^j$ increases the variation of the residuals e_i^j should increase as well. Thus, a plot of the residuals

e_i^j vs. $\mathcal{A}_i^j$ for $i = 1, \ldots, N^j$ should have a fan shape in the non-constant variance case. In summary, if the data set $\{\mathcal{A}_i^j\}_{i=1}^{N^j}$ is non-constant variance generated, then proportional error model given by Eq (4) is true. If the distribution of the errors $\{\varepsilon_i^j\}_{i=1}^{N^j}$ is i.i.d. then a plot of the *relative residuals* $r_i^j = e_i^j / \mathcal{A}_i^j$ vs. t_i^j for $i = 1, \ldots, N^j$ should exhibit a random pattern for non-constant variance generated data. A plot of r_i^j vs. $\mathcal{A}_i^j$ for $i = 1, \ldots, N^j$ should also exhibit a random pattern.

References

[1] Banks HT, Davidian M, Samuels JR, Sutton KL. An Inverse Problem Statistical Methodology Summary. In: Chowell G, Hyman JM, Bettencourt LMA, Castillo-Chavez C, editors. Mathematical and Statistical Estimation Approaches in Epidemiology. Dordrecht: Springer Netherlands; 2009. p. 249–302. Available from: `https://doi.org/10.1007/978-90-481-2313-1_11`.

[2] Marušić M, Vuk-Pavlović S. Prediction power of mathematical models for tumor growth. Journal of Biological Systems. 1993;01(01):69–78. doi:10.1142/S0218339093000069.

[3] Greene WH. Econometric Analysis. Pearson; 2012. Available from: `1161000/bf184dfa218e5a1839442ecfad2735bd`.
