# Supplementary material for: Parameter estimation and mathematical modeling for the quantitative description of therapy failure due to drug resistance in gastrointestinal stromal tumor metastasis to the liver
Source: PLoS One. 2019 May 30;14(5):e0217332. doi: 10.1371/journal.pone.0217332 (PMC6542538; doi:10.1371/journal.pone.0217332)
Supplement: S2 Appendix — (PDF) [file pone.0217332.s002.pdf]

S2 Appendix: Numerical method

In general the problem

$$\min_{\theta^j \in \mathbb{R}^8} S(\theta^j) + \kappa \|\theta^j\|_2^2 \,, \tag{1}$$

with $\kappa = 0$, *i.e.* the non-linear least squares problem, may have several local minima or may even not have a global minimum. This is a common issue when working with non-coercive functions as it usually occurs in inverse problems. This leads to an *ill-posedness* of the problem as discussed in [1]. This is a relevant issue when dealing with noisy-data, which is the case for the system addressed in this work. This justifies the introduction of the regularization term in the problem in Eq (1) with $\kappa > 0$. This problem has two important and interesting properties:

1. Under reasonable assumptions (continuity of the function $S(\cdot)$), the problem in Eq (1) has a unique optimal solution for any $\kappa > 0$ (see [2]). In contrast, for $\kappa = 0$ there are no general results on the existence of optimal solutions.

2. In the limit as the noise level λ tends to zero, *i.e.*, as the data quality becomes perfect, the solution of the problem with noise $\theta^j_{\lambda,\kappa}$ tends to the actual solution θ^j_κ in Eq (1).

Therefore, the parameter $\kappa > 0$ should be carefully chosen. If κ is too small, the propagation error due to data noise may become large; if κ is too big, the approximation error due to the weight of the term $\kappa\|\theta^j\|^2$ may become large. Sophisticated methods to obtain the best κ are based on the so-called *parameter choice strategies* such as Morozov discrepancy principle and the L-curve method, among others, which are beyond the scope of this work. For more details on these strategies and general regularization techniques, interested readers may refer to [1, 3].

We have numerically solved the optimization problem in Eq (1) for both sums of squares defined by

$$S(\theta^j) = \sum_{i=1}^{N^j} \left(\mathcal{A}_i^j - P(t_i^j, \theta^j)\right)^2 \tag{2}$$

$$S(\theta^j) = \sum_{i=1}^{N^j} \left(\frac{\mathcal{A}_i^j - P(t_i^j, \theta^j)}{\mathcal{A}_i^j}\right)^2 \,. \tag{3}$$

for several values of κ small enough, and found the best results for $\kappa = 0.001$. Since the objective functions defined by Eqs (2)-(3) have several local minima, a standard gradient type algorithm fails to converge, therefore in order to solve the problem in Eq (1) we chose a *direct-search* method, which does not utilize any approximation of the derivatives, but only evaluations of the objective functions. Specifically, we have applied the *Nelder-Mead simplex algorithm*, a very popular method for non-linear unconstrained optimization in many fields of science and technology, including biology and medicine [4]. This method is designed for unconstrained optimization problems with non-linear scalar objective functions and it has been widely utilized in statistics related optimization problems, particularly in the context of parameter estimation for non-linear deterministic models. For this kind of problems, the uncertain character comes from the noisy nature of the data, therefore statistical techniques is a natural choice to solve them.

Despite the popularity of the Nelder-Mead simplex algorithm in the scientific community, there are few results regarding its theoretical properties, such as convergence to actual optimal solutions. However, its introduction in the popular mathematical software Matlab® as the default algorithm, namely *fminsearch* solver, to perform minimization of scalar valued functions (see [5]), leaves this algorithm in the top of the recommended methods for numerical non-linear unconstrained optimization. For a detailed description of the Nelder-Mead simplex algorithm we recommend [6, 7, 8].

In order to implement the numerical method previously described, the following task outline was utilized:

- *Initialization:* to compute an initial guess of the parameter vector θ_0 by means of a random sampling technique.

- *Resolution of the proposed models*: to solve the models by using a Matlab® solver.
- *Resolution of the parameter estimation problem:* to perform non-linear optimization of the problem in Eq (1) by using a global minimization Matlab® solver.

Initialization: the main difficulty for addressing this step in an accurate way lies in the fact that no *a priori* information on the actual model parameters is available for any of the five proposed models. This is due to the fact that these parameters do not represent our observable quantity in a direct manner, which is why they have to be identified. The optimization algorithm has to be initialized with a suitable starting parameter vector θ_0. So, the choice of θ_0 is crucial as numerical solvers in general are highly dependent on this initial guess. To address this difficulty, we propose the following procedure. First, a box for the feasible parameters θ is defined by an upper and a lower bound for each component of the vector θ:

$$\Theta_{ad} = [(\mu_{MAX})_{\min}, (\mu_{MAX})_{\max}] \times [(\delta_{MAX})_{\min}, (\delta_{MAX})_{\max}] \times \ldots \times ([\nu_{\min}, \nu_{\max}] \text{ or } [\zeta_{\min}, \zeta_{\max}])\,.$$

It is worth noting that the intervals considered in the set Θ_{ad} previously defined, are chosen to keep the system's response within a biologically feasible range. Once Θ_{ad} is defined, a random sample of size n uniformly distributed in the previously defined set is selected, by using the so-called Latin Hypercube Sample technique (see [9]) implemented through the Matlab® tool *lhsdesign*, and the direct problem for each of the n obtained random parameters $\tilde{\theta}_i$ is solved. Finally, $\theta_0 = \arg\min_{1\le i\le n} S(\tilde{\theta}_i)$ is selected, where $S(\theta^j)$ is defined by Eqs (2) or (3).

Resolution of the proposed models: this was carried out with the stiff-ODE system solver *ode15s* from Matlab®. This is a variable-step, variable-order solver specially designed for stiff problems (see [10] for details); in our case, this solver was chosen since the models proposed often have stiff terms associated to the sampling of the random parameters $\theta_i \in \mathbb{R}^8$ for $i = 1, \ldots, n$ performed in the previous step.

Resolution of the parameter estimation problem: finally, to perform the minimization of the non linear problem given in Eq (1), we utilize the Nelder-Mead simplex algorithm implemented in Matlab® under the *fminsearch* subroutine.

The following summarizes the numerical implementation of the methods discussed above:

1. Load the data $(t_i^j, \mathcal{A}_i^j)$ for $i = 1, \ldots, N^j$, the initial conditions and the previously saved $\hat{\theta}^j$ for $j = 1, 2$ (optimal parameters from the last execution of the algorithm).
2. Set n (size of the random samples) and Θ_{ad} (feasible parameters).
3. Sample randomly the initial parameters $\tilde{\theta}_i^j \in \Theta_{ad}$, $i = 1, \ldots, n$, $j = 1, 2$.
4. Define θ_0^j as $\theta_0^j := \arg\min_{1\le i\le n} S(\tilde{\theta}_i^j)$.
5. Compare $S(\theta_0^j)$ with $S(\hat{\theta}^j)$. If $S(\theta_0^j) < S(\hat{\theta}^j)$ solve the problem in Eq (1) via the *fminsearch* subroutine, starting from the initial parameters θ_0^j. Else, break.
6. Define $\hat{\theta}^j$ as the output of the solver and save.

The algorithm was applied for each dataset and for each objective function (see Eqs (2) and (3)) separately.

References

[1] Engl HW, Flamm C, Kügler P, Lu J, Müller S, Schuster P. Inverse problems in systems biology. Inverse Problems. 2009;25(12):123014.

[2] Brezis H. Analyse fonctionnelle. Collection Mathématiques Appliquées pour la Maîtrise. [Collection of Applied Mathematics for the Master's Degree]. Paris: Masson; 1983.

[3] Engl HW, Hanke M, Neubauer A. Regularization of inverse problems. vol. 375. Springer Science & Business Media; 1996.

[4] Nelder JA, Mead R. A simplex method for function minimization. The computer journal. 1965;7(4):308–313.

[5] MATLAB Optimization Toolbox; 2010.

[6] Lagarias JC, Reeds JA, Wright MH, Wright PE. Convergence properties of the Nelder–Mead simplex method in low dimensions. SIAM Journal on optimization. 1998;9(1):112–147.

[7] Press WH. Numerical recipes 3rd edition: The art of scientific computing. Cambridge university press; 2007.

[8] Walters F, Parker Jr L, Morgan S, Deming S. Sequential Simplex Optimization. 1992. CRC Press, Boca Raton, Florida, USA 2l Jones AJ Genetic Algorithms and their Application to the design of Neural Networks Neural Computation and Applications. 1993;1(1):32–45.

[9] Iman RL, Campbell J, Helton J. An approach to sensitivity analysis of computer models. I-Introduction, input, variable selection and preliminary variable assessment. Journal of quality technology. 1981;13:174–183.

[10] Shampine LF, Reichelt MW. The MATLAB ODE suite. SIAM journal on scientific computing. 1997;18(1):1–22.
